# Acceptability of impregnated school uniforms for dengue control in Thailand: a mixed methods approach

**DOI:** 10.3402/gha.v7.24887

**Published:** 2014-09-01

**Authors:** Natasha Murray, Suphachai Jansarikij, Phanthip Olanratmanee, Pongsri Maskhao, Aurélia Souares, Annelies Wilder-Smith, Pattamaporn Kittayapong, Valérie R. Louis

**Affiliations:** 1 Institute of Public Health, University of Heidelberg, Heidelberg, Germany; 2 Center of Excellence for Vectors and Vector-Borne Diseases, Faculty of Science, Mahidol University, Salaya, Nakhon Pathom, Thailand; 3 Faculty of Humanities and Social Sciences, Rajabhat Rajanagarindra University, Chachoengsao, Thailand; 4 Lee Kong Chian School of Medicine, Nanyang Technological University, Singapore; 5 Department of Public Health and Clinical Medicine, Umeå University, Umeå, Sweden

**Keywords:** dengue, insecticide-treated material, permethrin, school uniforms, prevention, acceptability of impregnated school uniforms

## Abstract

**Background:**

As current dengue control strategies have been shown to be largely ineffective in reducing dengue in school-aged children, novel approaches towards dengue control need to be studied. Insecticide-impregnated school uniforms represent an innovative approach with the theoretical potential to reduce dengue infections in school children.

**Objectives:**

This study took place in the context of a randomised control trial (RCT) to test the effectiveness of permethrin-impregnated school uniforms (ISUs) for dengue prevention in Chachoengsao Province, Thailand. The objective was to assess the acceptability of ISUs among parents, teachers, and principals of school children involved in the trial.

**Methodology:**

Quantitative and qualitative tools were used in a mixed methods approach. Class-clustered randomised samples of school children enrolled in the RCT were selected and their parents completed 321 self-administered questionnaires. Descriptive statistics and logistic regression were used to analyse the quantitative data. Focus group discussions and individual semi-structured interviews were conducted with parents, teachers, and principals. Qualitative data analysis involved content analysis with coding and thematic development.

**Results:**

The knowledge and experience of dengue was substantial. The acceptability of ISUs was high. Parents (87.3%; 95% CI 82.9–90.8) would allow their child to wear an ISU and 59.9% (95% CI 53.7–65.9) of parents would incur additional costs for an ISU over a normal uniform. This was significantly associated with the total monthly income of a household and the educational level of the respondent. Parents (62.5%; 95% CI 56.6–68.1) indicated they would be willing to recommend ISUs to other parents.

**Conclusions:**

Acceptability of the novel tool of ISUs was high as defined by the lack of concern along with the willingness to pay and recommend. Considering issues of effectiveness and scalability, assessing acceptability of ISUs over time is recommended.

Dengue fever places a significant socio-economic and disease burden on many tropical and sub-tropical regions of the world, with children notably vulnerable to the disease (1–3). There is no vaccine or specific antiviral treatment available for dengue currently (4). Control of the disease therefore focuses on effective vector control methods; however, current vector control strategies have had very limited impact on the control of dengue to date (4, 5). Novel approaches for vector control have been called for.

Practical, acceptable, and community-based measures are urgently needed, particularly to protect vulnerable children at risk of dengue infection. *Aedes* mosquitoes mainly bite during the day. Because children spend most of their day at school, it has been suggested that preventive strategies should target schools and school activities (6). Schoolchildren in most endemic countries wear school uniforms on a daily basis during school times. A recent review on the safety and effectiveness of the use of insecticide-treated clothing indicated that it is a promising intervention (7). A recent mathematical modelling study showed that the use of insecticide-treated school uniforms could reduce the incidence of dengue infection up to 55% among school children (8). It has also been proposed to be a cost-effective strategy (9). To address this need specifically in children, the DengueTools consortium has initiated a double-blind, cross-over design, randomised controlled trial (RCT) to assess whether permethrin-impregnated school uniforms (ISUs) can reduce dengue incidence in Chachoengsao Province, Thailand (6, 10, 11). Ten schools, each with 100–500 students, are participating in the ongoing trial. As impregnated school uniforms have not been tested before in Thailand, exploring the acceptability of this novel approach is essential to assess scalability of ISUs in the future.

## Methodology

**Fig. 1:**
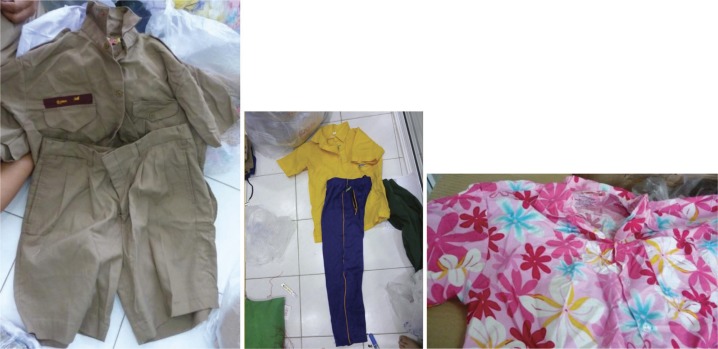
Example of school uniforms: standard, sport, and culture uniform.

A mixed methods approach was used to enable triangulation of both methods and data (12). The study was conducted in Chachoengsao Province, Thailand, in June and July of 2012, at the start of the RCT described in more detail by Wilder-Smith et al. (11). The intervention was randomised by schools. Students registered in a given school all received the same type of uniforms (treated or untreated). As the trial was double-blinded, neither the investigators, teachers, parents nor the students were informed whether the allocation was in the insecticide-treated school uniform group or the untreated school uniform group. To respect the RTC double-blind design, all uniforms were collected and sent to a factory (a subsidiary of Insect Shield International, LLC, Greensboro, NC, USA), irrespective of their ultimate treatment with permethrin or absence thereof. The photographs show examples of different school uniforms from this study (regular uniform, sports uniform, and culture uniform) (Fig. 1). Quantitative and qualitative data were collected concurrently from parents, teachers, and principals of students enrolled in the RCT (11). Ethical approval was gained from the University of Heidelberg as well as the Mahidol University Institutional Review Board (MUIRB) and conformed to the principles of the Declaration of Helsinki.

### Quantitative components

The study was conducted 4 weeks following initiation of the RCT, at which time four schools had students enrolled. Students enrolled in the RTC ranged in age from 7 to 18. We applied a school-proportional, class-clustered, randomised sub-sample of students enrolled. The inclusion criteria were parents of any student enrolled in the RCT, who had been wearing the ‘trial’ uniforms for at least 4 weeks. Parents completed self-administered in-depth questionnaires to establish background socio-demographic information, knowledge, and experience of dengue and assess the acceptability of ISUs including concerns, willingness to pay, and willingness to recommend. A research assistant with comprehensive knowledge of Thai culture, both the English and Thai languages, as well as background knowledge of the RCT and the objectives of this study translated the questionnaire into Thai. The questionnaire was then ‘back-translated’ into English to assess for inconsistencies and allow for correct interpretation of responses at the time of analysis (13, 14). All parents were provided an information sheet and consent form for participation in the study.

Microsoft^®^ Excel^®^ for Mac 2011 (Version 14.0.0 – 100825) and SAS Version 9.2 were used for data input and analysis. All confidence intervals (CIs) displayed were calculated at 95%. Multivariate analysis was performed to identify which independent variables maintained a significant association with ‘willingness to pay additionally’. A strategy of manual backward selection was used, starting with the model including all covariates that were significant with univariate analysis. At each step, the most non-significant covariate (based on Wald statistics) was removed from the model. The SAS procedure LOGISTIC was run for the logistic regression model using categorical descriptive variables with ‘yes’ as event for the output variable.

### Qualitative components

Qualitative components included focus group discussions (FGDs) and individual semi-structured interviews involving parents and teachers from the same four schools where quantitative data were gathered. Four FGDs were conducted with a purposive sampling of about 5–8 parents and teachers of students enrolled in the RCT to gain a deeper perspective on the group societal and cultural perspectives and norms (15). Key informant interviews were undertaken with two school principals, due to their decision-making influence regarding implementing novel tools in their schools, such as ISUs. Prior to conducting the qualitative components, a researcher from a local institution reviewed both the FGD and interview templates. Following translation into Thai, the templates were back-translated into English to ensure key elements to meet study objectives remained.

A trained Thai researcher conducted the FGDs and interviews using this pre-defined template. Data were collected during the FGDs and interviews by both the moderator/interviewer and a note taker, who documented all quotations. The two collaborated on responses following each FGD/interview. The de-identified transcription was initially developed in Thai, followed by translation into English.

English-translated transcripts from the FGDs and interviews were analysed manually and structured with the principles of content analysis using coding and thematic development. Codes were developed both deductively and inductively. In order to enhance the reliability of the analysis (15), a trained qualitative researcher independently coded the de-identified transcripts. The two coding schemes were then reviewed, discussed, and developed into the final coding scheme. Finally, themes and sub-themes identified were presented concurrently with quantitative results for transparency and enhancing triangulation of methods.

Open responses to the quantitative in-depth questionnaire for parents were analysed by the qualitative methods described and integrated with other qualitative data.

## Results

### Socio-demographic background

Basic socio-demographic information of the sample of parents who completed self-administered in-depth questionnaires is summarised in Table 1. Parents of students from grades one to seven were represented. Among respondents, 5.9% had no education, 55.1% had completed primary school, and the remainder had completed secondary school or above. The total monthly income of households was variable with 27.1% earning less than 5,000 Baht and 60.7% earning less than 10,000 Baht (100 Baht≈3USD). The majority of respondents were employed by others (70.4%); 66.7% owned their house, and 16.2% stayed in rented accommodation.

**Table 1:** Basic socio-demographic and household information from quantitative parent questionnaires (N=321)

	n	%
Student grade		
Grade 1	64	20.0
Grade 2	27	8.4
Grade 3	34	10.6
Grade 4	26	8.1
Grade 5	42	13.1
Grade 6	113	35.2
Grade 7	15	4.7
Highest level of education of respondent		
None	19	5.9
Primary school	177	55.1
Secondary school	61	19.0
Higher certificate of education	14	4.4
University diploma	12	3.7
University bachelor degree	8	2.5
University masters or above	1	0.3
Missing data	29	9.0
Total monthly income of household in Thai Baht (100 Baht≈3USD)		
0–4,999	87	27.1
5,000–9,999	108	33.6
10,000–14,999	54	16.8
15,000–19,999	24	7.5
20,000 or above	23	7.2
Missing data	25	7.8
Employment status of respondent		
Employed (by someone other than self)	226	70.4
Self-employed	43	13.4
Unemployed	37	11.5
Missing data	15	5.7

### Knowledge and experience of dengue and vector control tools

#### Quantitative

Parents (17.3%; 50/289, 95% CI 13.1–22.1) stated they had experienced at least one household member having dengue in the previous 12 months and there was predominantly moderate to high level of concern of dengue in the district (Table 2). Parents (77.1%; 236/306, 95% CI 72.0–81.7) deemed dengue to be of severe danger or life threatening (Table 2).

**Table 2:** Frequency and percentages of danger of dengue and level of concern of dengue in district among parents (N=321)

	n	%	% range amongst schools
Level of concern of dengue in district			
None	34	10.6	[3.3–15.0]
Mild	30	9.3	[3.7–13.4]
Moderate	145	45.2	[35.5–50.8]
High	99	30.8	[19.4–42.5]
Missing data	13	4.1	[0–9.0]
Danger level of dengue			
Not dangerous at all	6	1.9	[0.9–2.8]
Slightly dangerous	33	10.3	[8.4–17.5]
Mildly dangerous	6	1.9	[0–3.7]
Moderately dangerous	25	7.8	[2.8–11.2]
Severely dangerous/life threatening	236	73.5	[67.1–77.5]
Missing data	15	4.7	[0–12.0]

#### Qualitative Knowledge of dengue

The general knowledge of dengue was similar between teachers, parents, and principals who participated. All noted the disease is caused by the bite of a mosquito. There was even specific knowledge of the term ‘Aedes’ mosquitoes and day-time biting by some of the participants:

Mosquito, Aedes mosquito. It can be found during the day time. (G6 Parent, FDG, School B)

The following sites where ‘Aedes’ mosquitoes could be located were identified: water pipes, dirty water, forest, plant pots, and in dark corners. Participants also noted that some buildings do not have any mosquitoes. The majority of participants correctly identified symptoms of dengue, such as fever and rash. Both children and adults were described as being at risk of dengue fever and children more specifically identified at higher risk of complications. One principal stated the following when questioned on their opinion of the RCT:

I thought this project is useful and it helps children because children normally get infected with dengue …. If it benefits students who have a risk to be dead or may die from the disease …. (Principal, Interview)

Further details of dengue were described by both teachers and principals. For example, a teacher made reference to the number of dengue virus strains.

I know that dengue has four different strains. (Grade four Teacher, FGD)

Participants of FGDs and interviews identified multiple known and used vector control tools for dengue (Table 3). These have been classified into environmental management tools, chemical control tools, biological control tools, and others.

**Table 3:** Known and used vector control tools of school teachers, parents, and principals participating in FGDs and interviews

Classification of vector control tool	Known and used vector control tools
Environmental	Water jar lids/seals and water container lids
management	Turn over water containers
	Wire screen
	Changing of water
	Remove breeding sites
	Destroy unused containers
	Bed net
	Keeping house and surrounds clean
	Ventilation
	Fill water hole with soil
Chemical control	Temephos
	Mosquito coils and sticks
	Fogging – at home and schools
	Insecticide at home
	Insecticide spraying by municipality in schools
	Personal repellent
Biological control	Guppy fish
Other methods identified	Education of students and public relations campaigns
	Posters about dengue prevention
	Adding Kafir lime to drinking water
	Electric racket

### Acceptability of ISUs – concerns regarding ISUs

#### Quantitative

As a general assessment of whether any concerns would restrict parents from allowing their students to wear ISUs independent of this trial, parents were asked if they would allow their child to wear an ISU if it was provided free of charge. In response, 87.3% (260/298, CI 82.9–90.8) reported they would, 10.1% (30/298, CI 6.9–14.1) were uncertain, and 2.7% (8/298, CI 1.2–5.2) stated they would not.

#### Qualitative

Participants were asked to present any concerns (or lack thereof) with regards to allowing their children or students to wear ISUs. A variety of responses were provided and discussed.

Concerns expressed by participants included the limited physical coverage of trial uniforms due to them being short sleeved and shorts/skirts with exposed skin on legs and arms. Individuals experienced children being bitten on the legs and concern arose about protection of the exposed body parts.

The uniform can only protect the ‘body’ part, not the arms and legs. (Grade three Teacher, FGD)

Teachers also questioned the effect of the days the children do not wear uniforms, for example, when they stay at home or non-school days.

If students stay at home, how can it work? (Grade seven Teacher, FGD)

The practicalities of children growing out of their ‘impregnated clothing’ were highlighted by one parent.

It's not practical because a shirt is too small to use when the child grows up. (Grade six Parent, FGD)

Concerns regarding allergies were discussed in FGDs and interviews. This was predominantly with regard to initial concerns of parents being alleviated by a pre-trial experience with insecticide-treated wrist-bands.

No concerns at all. Because there is no allergic reaction after using treated wrist-band. (Grade four Teacher, FGD)

Many participants responded by stating they had ‘no concerns’ with allowing their children to wear ISUs and a range of reasons for this was provided.

No concerns. Students behave as usual. (Grade four Teacher, FGD)

Uniform is not dangerous. (Grade two Parent, FGD)

This method can protect my children from mosquito bite. (Grade three Teacher, FGD)

### Acceptability of ISUs – willingness to pay

#### Quantitative

Parents were asked how much they would be willing to pay additionally for an ISU over a ‘normal’ school uniform (Table 4). Parents (40.1%; 105/262, CI 34.1–46.3) were not willing to pay anything additional to purchase an impregnated uniform, and the remainder were willing to pay differing amounts up to and over 1,000 Baht (157/262, CI 53.7–65.9). Willingness to pay was significantly associated with higher level of education of the respondent and the total monthly income of the household (Table 5).

**Table 4:** Willingness to pay additionally among parents to purchase an impregnated uniform over a ‘normal’ school uniform (100 Baht≈3USD)

	Not willing	Willing	Total
	
Category	n (%)	n (%)	n (%)
Amount (Baht) (N=321)			
0			105 (32.7)
1–200			108 (33.6)
201–400			39 (12.2)
401–600			4 (1.3)
601–800			3 (0.9)
801–1,000			0 (0)
1,000 & above			3 (0.9)
Missing			59 (18.4)
Education level (N=240)			
None	8/96 (8.3)	8/144 (5.6)	16/240 (6.7)
Primary school	67/96 (69.8)	74/144 (51.4)	141/240 (58.8)
Secondary school and above	21/96 (21.9)	62/144 (43.1)	83/240 (34.6)
Monthly income (N=246)			
0–4,999	35/98 (35.7)	31/148 (21.0)	66/246 (26.8)
5,000–9,999	38/98 (38.8)	53/148 (35.8)	91/246 (37.0)
10,000–14,999	14/98 (14.3)	36/148 (24.3)	50/246 (20.3)
15,000 & above	11/98 (11.2)	28/148 (18.9)	39/246 (15.8)

**Table 5:** Logistic regression model of willingness to pay additionally (yes/no) regressed against total monthly income of household (grouped as 0–4,999, 5,000–9,999, and ≥10,000 Baht) and education level of respondent (grouped as none, primary, and secondary and above)

Parameter	Estimate	Error	Odd ratio	95% CI
Intercept	−1.557	0.600		
Monthly income	0.368	0.184	1.44	1.01–2.07
Education level	0.529	0.257	1.70	1.03–2.81

#### Qualitative

Parents were asked about their willingness to pay additionally for the ISUs. Teachers and principals were asked to provide a recommendation of the amount parents should pay additionally for the ISUs.

Initial response to these questions varied with the majority of participants stating they should be provided free of charge. With further probing, some stated a Thai Baht value they would be willing to pay, and others stated factors that the amount of payment should be dependent on.

Most participants felt that the impregnation of uniforms should hold no additional cost and furthermore, some thought the government should provide them.

I think it should have no cost at all. Government should do them for free before they give them to the students. (All Grades Teacher, FGD)

After probing for responses, some participants indicated values they would be additionally willing to pay for impregnation of uniforms, ranging between 30 and 50 Baht per item of clothing. A range of 30–50 Baht per item was agreed upon in each FGD.

Teachers and parents indicated that the additional cost to be placed on impregnation of student's uniforms should be dependent on a family's ability to pay, not on their willingness to pay as some may not have the additional funds.

Depends on the finance of each family. (Grade four Teacher, FGD)

Depends on each family but most of them are poor. (All Grades Teacher, FGD)

Parents also questioned the length of time the impregnation lasted for and stated this would alter their willingness to pay.

Depend on how long is the lifetime. (Grade six Parent, FGD)

One principal indicated they would like to see the results of the trial prior to stating a reasonable fee to pay additionally for impregnation. When asked their opinion in the situation where the trial showed uniforms to be effective in reducing dengue fever, they felt there should be no additional cost for impregnation, reflecting on the wider impact on health and governmental healthcare savings.

Price should be no different than normal clothes. It should not be expensive because it will be used for health. If we use this budget for prevention, it could help the government save the cost of medical care during patient admission. (Principal, Interview)

### Acceptability of ISUs – willingness to recommend

#### Quantitative

The majority of parents indicated they would be willing to recommend ISUs to other parents (62.5% [180/288, CI 56.6–68.1]). Parents (9.4%; 27/288, CI 6.3–13.4) said they would not, and 28.1% (81/288, CI 23.0–33.7) were uncertain.

#### Qualitative

When parents were asked directly about their willingness to recommend ISUs and how they would recommend them to other parents, those who were willing primarily discussed the protective and beneficial effect for their children, based on their ideas and experiences. The statements they would make to other parents included:

I feel good. I feel like this project helps us take care of our children and teach us about the caution. (Grade six Parent, FGD)

Can prevent mosquito bite. (Grade six Parent, Open Response)

Some parents made reference to awaiting results of the trial prior to a decision on recommending to other parents. They specified recommendation being based on efficacy and safety of the ISUs, with no side effects or allergy.

If it is good, I will pass along. (Grade six Parent, Open Response)

If there are no side effect. (Grade two Parent, Open Response)

Some parents stated they were not willing to recommend ISUs to other parents. Reasons included being unaware of how to explain ISUs to others, and the concern that they would be blamed if they do recommend and another person's child has an allergic reaction to the ISU, again indicating concerns with allergies.

## Discussion

Knowledge and awareness about dengue was found to be high, consistent with findings of previous studies in Thailand (16–18). The knowledge displayed was profound not only regarding the cause of dengue but also vector control. Participants in qualitative components were able to detail the transmitting vector, the infected mosquito and some even further identified the *Aedes* mosquito specifically. The significantly relevant day-time biting activities of the mosquito were mentioned by participants and some even discussed the applicable use of ISUs to target this biting activity pattern. Common sites where *Aedes* mosquitoes are found were described well, as with other research findings from Thailand (18, 19). Symptoms of dengue and the increased burden of disease on children over adults were identified by teachers, parents and principals. However, only teachers and principals gave a more detailed description of the viral strains of dengue. In a study by Van Benthem et al. (18) in Northern Thailand, a ‘good’ knowledge of dengue was classified by knowledge of one symptom of the disease, determined in 67% of their sample. Based on these criteria, we can also classify a ‘good’ level of knowledge in our purposive samples, as multiple symptoms were identified amongst participants. The two common symptoms identified in both this and Van Benthem et al. (18) research were fever and rash. Knowledge and practices of vector control were also noted extensively in FGDs and interviews by parents, teachers and principals alike, in accordance with previous studies on vector control in Thailand (18, 19). Participants mentioned using all the different forms of environmental, chemical, and biological vector control tools in their households, as well as some being distributed by the municipality. Principals and teachers also discussed how children are educated on methods to use at school and at home, as to further practices of vector control.

With regard to experience of dengue illness in households, 17% of parents indicated that at least one household member had suffered from dengue in the previous 12 months. Moreover, participants expressed a high level of concern of dengue in their district and were all aware of the potentially life-threatening outcomes of dengue. Such substantial direct dengue experience and awareness regarding the severity and health impact explains the high perceived concern in Thailand. This has also been identified previously as a reason for the extensive use of vector control techniques to prevent disease (19). This underpins both the parents’ and teachers’ eagerness to explore novel dengue control methods such as ISUs.

Not unsurprisingly then, we found high acceptability of ISUs amongst parents, teachers, and principals assessed in the early stages of the RCT. This was reflected by a lack of concern and the presence of willingness to pay and recommend. Of those who expressed some uncertainty, the majority related this to awaiting results of the trial on whether ISUs have efficacy in their desired purpose. Very few respondents indicated negative factors that could potentially be associated with lack of acceptability. Hence, 87% of parents indicated they would allow their student to wear an ISU if freely provided. Over 50% of parents were even willing to pay additionally for ISUs over normal school uniforms. The specific amount that the majority of parents were willing to pay was less than 200 Baht. Correspondingly, 30–50 Baht per piece (60–100 Baht total) was agreed upon in qualitative FGDs and interviews. However, reservations about additional payments were also articulated during the FGDs. Multiple participants insisted that the government should pay any additional expenses of impregnation, in the situation where impregnated ISUs are shown to be an effective tool for dengue control. The participants felt that it would be the government's responsibility to ensure good dengue control. This idea was developed further by one principal who related the cost-effectiveness of ISUs for the government in preventing disease and admissions, should they be shown to be effective.

Further discussed in qualitative components was that some families would not be able to afford to pay for ISUs, an idea that has been referred to in previous ITM studies and raises the impact of ability to pay on willingness to pay (16, 20). The correlation of ‘ability to pay’ affecting willingness to pay can be inferred from the regression analysis, which showed a higher willingness to pay with an increasing total monthly income of the household. As a means of addressing an ability to pay to increase community availability of vector control tools, subsidisation or provision to those who are unable to afford them has been suggested in other ITM studies (21).

Higher educational level was also significantly linked with willingness to pay. A similar pattern was evident in a study on Long Lasting Insecticidal Nets (LLINs) in Orissa, India (22). In this setting, however, 50–78% of respondents had a background of no education and those with primary or higher education were shown to be significantly more willing to pay for LLINs. In contrast, the current sample included only 5.9% who had no education. This reflects the high level of at least primary education in the Thai population, consistent with the Ministry of Education's principle of ‘lifelong’ education and compulsory nine years of primary schooling (23).

Finally, we looked at willingness to ‘recommend’ ISUs. We found that 63% of parents were willing to recommend ISUs to other parents, few were not (9%) and some were uncertain at the time of assessment (28%). In a study of insecticide-treated curtains and jar covers in Venezuela, the willingness to recommend to friends and relatives was also high at 78.8% when initially assessed 6 months into the trial (16). However, this result had reduced to 59.1% by the end of their trial, suggesting that although the results on willingness to recommend ISUs are currently promising, continual review is warranted throughout the RCT to assess for changes. Uncertainty of willingness to recommend was portrayed in both quantitative and qualitative elements of this study, with the common theme of desire to await efficacy, safety and allergy results of the trial prior to recommending to others.

One limitation of the study was that it was performed at the very beginning of the trial and hence we cannot describe the perception and acceptance as it may evolve over time with real-life experiences further on in the trial. Another limitation in the quantitative section was the use of self-reporting questionnaire among parents of students enrolled in the study. This may have led to a selection bias of the returned questionnaires. Despite these limitations and thanks to the mixed methods design, this study provides valuable information, within a blinded methodology, about the initial attitude towards the use of impregnated clothing.

In conclusion, this study provided insights into the acceptability of a novel dengue control tool based on ISUs. With a mixed-method approach, a robust understanding of the perception of ISUs in the Thai context was obtained. As the factory-impregnation of the school uniforms could not be detected via smell or other changes in the textiles, with no obvious reactions to these uniforms found in the first 4 weeks of the trial, the extent of concern about ISUs was low. We did not identify major barriers that would hinder routine use of ISUs in the future. Based on the high acceptability of ISUs found both in the quantitative and qualitative elements of this study, ISUs could potentially be rolled out as a novel intervention should the trial show efficacy in reducing dengue incidence in school-aged children.

## References

[1] Guzman MG, Kouri G (2002). Dengue: an update. Lancet Infect Dis.

[2] Gubler DJ (2011). Dengue, urbanization and globalization: The Unholy Trinity of the 21(st) Century. Trop Med Health.

[3] Murray NE, Quam MB, Wilder-Smith A (2013). Epidemiology of dengue: past, present and future prospects. Clin Epidemiol.

[4] WHO (2009). Dengue: guidelines for diagnosis, treatment, prevention and control – New edition.

[5] Morrison AC, Zielinski-Gutierrez E, Scott TW, Rosenberg R (2008). Defining challenges and proposing solutions for control of the virus vector Aedes aegypti. PLoS Med.

[6] Wilder-Smith A, Lover A, Kittayapong P, Burnham G (2011). Hypothesis: impregnated school uniforms reduce the incidence of dengue infections in school children. Med Hypotheses.

[7] Banks SD, Murray N, Wilder-Smith A, Logan JG (2014). Insecticide-treated clothes for the control of vector-borne diseases: a review on effectiveness and safety. Med Vet Entomol.

[8] Massad E, Amaku M, Coutinho FA, Kittayapong P, Wilder-Smith A (2013). Theoretical impact of insecticide-impregnated school uniforms on dengue incidence in Thai children. Glob Health Action [Research Support, Non-U.S Gov't].

[9] Tozan Y, Ratanawong P, Louis VR, Kittayapong P, Wilder-Smith A (2014). Use of insecticide-treated school uniforms for prevention of dengue in schoolchildren: a cost-effectiveness analysis. PLOS one (in press).

[10] Wilder-Smith A, Renhorn KE, Tissera H, Abu Bakar S, Alphey L, Kittayapong P (2012). DengueTools: innovative tools and strategies for the surveillance and control of dengue. Glob Health Action.

[11] Wilder-Smith A, Byass P, Olanratmanee P, Maskhao P, Sringernyuang L, Logan JG (2012). The impact of insecticide-treated school uniforms on dengue infections in school-aged children: study protocol for a randomised controlled trial in Thailand. Trials.

[12] Padgett D (2012). Qualitative and mixed methods in public health.

[13] Mays N, Pope C (1995). Rigour and qualitative research. BMJ.

[14] Fourie J, Feinauer I (2005). The quality of translated medical research questionnaires. South Afr Ling Appl Lang Stud.

[15] Britten N (1995). Qualitative interviews in medical research. BMJ.

[16] Vanlerberghe V, Villegas E, Jirarojwatana S, Santana N, Trongtorkit Y, Jirarojwatana R (2011). Determinants of uptake, short-term and continued use of insecticide-treated curtains and jar covers for dengue control. Trop Med Int Health.

[17] Vanlerberghe V, Villegas E, Oviedo M, Baly A, Lenhart A, McCall PJ (2011). Evaluation of the effectiveness of insecticide treated materials for household level dengue vector control. PLoS Negl Trop Dis.

[18] Van Benthem BH, Khantikul N, Panart K, Kessels PJ, Somboon P, Oskam L (2002). Knowledge and use of prevention measures related to dengue in northern Thailand. Trop Med Int Health.

[19] Paz-Soldan VA, Plasai V, Morrison AC, Rios-Lopez EJ, Guedez-Gonzales S, Grieco JP (2011). Initial assessment of the acceptability of a Push–Pull Aedes aegypti control strategy in Iquitos, Peru and Kanchanaburi, Thailand. Am J Trop Med Hyg.

[20] Rowland M, Durrani N, Hewitt S, Mohammed N, Bouma M, Carneiro I (1999). Permethrin-treated chaddars and top-sheets: appropriate technology for protection against malaria in Afghanistan and other complex emergencies. Trans R Soc Trop Med Hyg.

[21] Seng CM, Setha T, Nealon J, Chantha N, Socheat D, Nathan MB (2008). The effect of long-lasting insecticidal water container covers on field populations of Aedes aegypti (L.) mosquitoes in Cambodia. J Vector Ecol.

[22] Gunasekaran K, Sahu SS, Vijayakumar KN, Jambulingam P (2009). Acceptability, willing to purchase and use long lasting insecticide treated mosquito nets in Orissa State, India. Acta Trop.

[23] Ministry of Education (2009). The Development and State of the Art Adult Learning and Education (ALE): National Report of Thailand.

[24] Jaenisch T, Sakuntabhai A, Wilder-Smith A (2013). Dengue research funded by the European Commission-scientific strategies of three European dengue research consortia. PLoS Negl Trop Dis.

